# A Novel Image Encryption Scheme Based on Elliptic Curves over Finite Rings

**DOI:** 10.3390/e24050571

**Published:** 2022-04-19

**Authors:** Umar Hayat, Ikram Ullah, Naveed Ahmed Azam, Sumaira Azhar

**Affiliations:** 1 Department of Mathematics, Quaid-i-Azam University, Islamabad 45320, Pakistan; ikram.ullah@math.qau.edu.pk (I.U.); 03141713011@student.qau.edu.pk (S.A.); 2 Department of Applied Mathematics and Physics, Graduate School of Informatics, Kyoto University, Kyoto 606-8503, Japan

**Keywords:** elliptic curve, finite ring, total order, substitution box, image encryption

## Abstract

Image encryption based on elliptic curves (ECs) is emerging as a new trend in cryptography because it provides high security with a relatively smaller key size when compared with well-known cryptosystems. Recently, it has been shown that the cryptosystems based on ECs over finite rings may provide better security because they require the computational cost for solving the factorization problem and the discrete logarithm problem. Motivated by this fact, we proposed a novel image encryption scheme based on ECs over finite rings. There are three main steps in our scheme, where, in the first step, we mask the plain image using points of an EC over a finite ring. In step two, we create diffusion in the masked image with a mapping from the EC over the finite ring to the EC over the finite field. To create high confusion in the plain text, we generated a substitution box (S-box) based on the ordered EC, which is then used to permute the pixels of the diffused image to obtain a cipher image. With computational experiments, we showed that the proposed cryptosystem has higher security against linear, differential, and statistical attacks than the existing cryptosystems. Furthermore, the average encryption time for color images is lower than other existing schemes.

## 1. Introduction

In this modern era, the transmission of images over public networks is an indispensable task. Therefore, distributing secret images in a secure way is essential. Encryption schemes take plain-text data as an input and convert it into an unreadable form using secret keys. Then, an authorized person uses the secret keys to acquire the original data. Recently, S-box-based encryption algorithms have gained special attention [1]. An S-box is used to create confusion; therefore, researchers are not only trying to improve the existing S-box schemes, but are also proposing new ones. Many authors (see, for example, [2,3]) have observed that the S-box used in the well-known cryptosystem, the advanced encryption standard (AES), is vulnerable because the AES cryptosystem uses a static S-box. Furthermore, it also has low algebraic complexity.

The role of an S-box is vital in a cryptosystem. Therefore, researchers are trying to generate S-boxes which are cryptographically secure. One way is to improve the security of the AES cryptosystem. That is why Cui and Cao [4] enhanced the algebraic complexity of an AES S-box to make it secure against algebraic attacks. Similarly, Liu et al. [5] proposed an improved AES S-box that has reliable security against algebraic and differential attacks. Tran et al. [6] presented the Gray S-box for the AES, which is secure against algebraic and interpolation attacks. In addition to this, many other techniques are used to design S-boxes with the desired security. For example, Silva-Garcia et al. [7] developed a novel chaos-based cryptosystem, which generates an S-box to resist linear attacks. Ibrahim and Alhabi [1] used a Henon map to generate secure and dynamic S-boxes. Özkaynak [8] proposed robust S-boxes using different chaotic systems. Due to their higher security, EC-based cryptosystems have gained the interest of researchers. Miller [9] presented an EC-based cryptosystem with high security and a small key size. Cheon et al. [10] used hyperelliptic curves to obtain a lower bound on the nonlinearity of Boolean functions. Hayat et al. [11,12] employed ECs over finite fields and rings to design secure S-boxes. It is analyzed that the S-box generators over finite rings generate dynamic S-boxes with reasonable time complexity.

Furthermore, the pixels have high correlation and the data size is large in an image. Therefore, to develop secure cryptosystems for the delivery of secret images is a common interest of researchers. Many techniques, such as fuzzy theory [13] and chaotic maps [14,15,16,17,18,19,20,21,22,23,24,25], are used to develop encryption schemes.

Recently, chaos-based schemes have received more attention due to their easy implementation and fast execution [26]. Similarly, chaotic maps have some unique characteristics, such as ergodicity, unpredictability, random behavior, and sensitivity to initial parameters, which are required for a good cryptosystem [27]. Therefore, a number of image encryption schemes are proposed based on sine maps [28,29], cat maps [30], and some other chaotic maps [31]. Huang et al. [32] developed an encryption scheme with better confusion-creation capability using the Rivest–Shamir–Adleman (RSA) algorithm and Arnold map. Similarly, Ye et al. [33] designed an effective and secure image cryptosystem based on the RSA technique and a fractional-order chaotic system. On the other hand, there exist chaotic encryption schemes which are not secure. Chen et al. [30] developed an image encryption algorithm using a 3D chaotic cat map, which has short period and hence, is vulnerable to the chosen plain-text attack [34]. Similarly, the scheme developed by Sui et al. [31] is based on logistic maps; however, logistic maps are insecure due to the small key space [14].

Related work: Like chaotic maps, elliptic curves (ECs) are highly sensitive to the initial parameters, and a number of EC-based algorithms have been developed for cryptographic applications [9,35,36,37,38,39,40,41,42,43,44,45,46,47,48]. Zhang and Wang [40] used the group law for the generation of a public key and encrypted digital images by combining a chaotic system and elliptic curve cryptography (ECC). Abdelfatah [41] presented a digital signature scheme using the group law. Abbas et al. [42] proposed a chaotic encryption algorithm using an addition operator over the points of the EC. El-Latif et al. [49] developed an efficient encryption scheme using a cyclic EC and a generalized logistic map; basically, an EC-based sequence and a chaotic sequence are combined to generate a keystream for encryption. Toughi et al. [50] generated a sequence of numbers using ECs, which are then used in the generation of keys to propose an encryption algorithm. Hayat et al. [51] proposed a twofold image encryption scheme. Firstly, a plain image is masked by EC-based random numbers, and then permuted by an S-box generated using an EC. Reyad et al. [52] modulated random sequences using ECs and chaotic maps. It is experimentally proved that all the above schemes are highly secure. In addition, ECs provide more security to a cryptosystem than chaotic maps [53,54], but it is important to mention that the scheme in [49] first generates a sequence of scalars. Each scalar is then mapped to a scalar multiple of a point of a cyclic EC. To compute a scalar multiple of a point lying on an EC is not simple; it involves the group law, consisting of many complex calculations. Similarly, the scheme in [52] also uses group operations to generate random numbers from the points of ECs. Thus, the schemes of [40,41,42,44,49,52] are time-consuming due to the use of the group law. The scheme in [51] avoids the group law to generate ECs, but each plain image requires the generation of two ECs. Moreover, trials are required for the generation of ECs to ensure the encryption of an image because the said scheme does not output an S-box for all parameters. These facts slow the execution time of the scheme. The algorithm in [37] does not generate triads for all images of the same size, but generates an EC for each image, which enhances the execution time.

In all the above-discussed EC-based schemes, finite fields are used to obtain the desired security. The security of such cryptosystems based on ECs over finite fields essentially depends on the computational cost for solving the discrete logarithm problem [55]. Recently, Diaz et al. [55] pointed out that the cryptosystems based on ECs over finite rings are more secure as compared to the cryptosystems based on ECs over finite fields. Their claim follows from the fact that the computational cost of breaking such cryptosystems based on ECs over finite rings depends on solving the factorization problem [56] and the discrete logarithm problem. Our research contribution aims to develop a cryptosystem that is based on a ring of integers and that has higher resistance against modern attacks than the existing schemes of ECs over finite fields. In this work, we propose:

1. A new S-box generator that can generate a good S-box based on an EC over a finite ring of integers;
2. An image encryption algorithm using the aforementioned S-box generator.

The current work is novel in the sense that:

- It is based on a ring of integers rather than on a finite field;
- It avoids the traditional way (group law) of generating an EC. Furthermore, it imposes a bound on the *y*-coordinate of the EC, which is not the case in [12,51], and one does not have to check all the possible values over the underlying structure. So, the current S-box generator is comparatively efficient for a possible S-box;
- It generates cryptographically strong S-boxes;
- The time complexity of the proposed scheme is dependent on the chosen bound on the *y*-coordinate of the EC;
- Unlike the case in [37,51,57], each input image does not need the computation of a new EC for the confusion phase;
- It can encrypt a number of images with better security against differential, statistical, key and plain-text attacks;
- The run-time of the proposed scheme to encrypt color images is very low.

The rest of the paper is organized as follows: Section 2 contains the related notions of ECs. In Section 3, the proposed S-box algorithm and its analysis is described. In Section 4, the proposed image encryption scheme is presented. In Section 5, the decryption of the proposed scheme is reported. The security analysis is conducted in Section 6. An application of the proposed scheme to color images is shown in Section 7. Lastly, the conclusion is drawn in Section 8.

## 2. Related Notions of ECs

Let ${\left\{p_{i}\right\}}_{i\in \Omega }$ be a set of primes for any indexing set Ω. Then, for k≥2, $n=\prod_{i=1}^{k}p_{i}$ is a composite integer and, hence, $Z_{n}$ is a ring. As for each prime *p*, $F_{p}$ represents a finite field. Thus, for primes $p_{i},1\leq i\leq k$, we say that $F_{p_{i}}$ is a finite field related to the ring $Z_{n}$. For any two integers, $a,b\in Z_{n}$, with the condition that $4a^{3}+27b^{2}\in Z_{n}\backslash \left\{0\right\}$, the EC $E_{n,a,b}$ represents the set of points $\{\left(x,y\right)\in Z_{n}\times Z_{n}|y^{2}\equiv x^{3}+ax+b\;\left(\mathrm{mod}\;n\right)\}\cup \left\{\infty \right\}$, where *∞* is the neutral point lying at each vertical line passing through the EC, and $4a^{3}+27b^{2}$ is the discriminant of $E_{n,a,b}$. The condition on the discriminant is imposed so that the EC $E_{n,a,b}$ has no singular point. The integers n,a and *b* are known as the parameters of $E_{n,a,b}$. For a=0, the ECs are known as Mordell elliptic curves (MECs); we denote them by $E_{n,b}$, and $\#E_{n,b}$ denotes the number of points on the MEC $E_{n,b}$. There is a bijection [56] between $E_{n,b}$ and $E_{p_{1},b}\times \ldots \times E_{p_{k},b}$, which maps $\left(x,y\right)\in E_{n,b}$ to $\left(x\;\left(\mathrm{mod}\;p_{1}\right),y\;\left(\mathrm{mod}\;p_{1}\right)\right)\times \ldots \times \left(x\;\left(\mathrm{mod}\;p_{k}\right),y\;\left(\mathrm{mod}\;p_{k}\right)\right)\in E_{p_{1},b}\times \ldots \times E_{p_{k},b}.$ Consequently, an EC $E_{p_{i},b}$ may be deduced from $E_{n,b}$ by mapping $\left(x,y\right)\in E_{n,b}$ to $\left(x\;\left(\mathrm{mod}\;p_{i}\right),y\;\left(\mathrm{mod}\;p_{i}\right)\right)$ and, hence, for each $p_{i}$ we have a surjective map from $E_{n,b}$ to $E_{p_{i},b}$. Let $f:E_{n,b}\to E_{p_{1},b}\times \ldots \times E_{p_{k},b}$ and $g:E_{n,b}\to E_{p_{i},b}$ represent the former bijective and surjective maps, respectively; then, mathematically, *f* and *g* are given by:

$$\begin{array}{c} \begin{array}{cc} f\left(x,y\right)=(x\;\left(\mathrm{mod}\;p_{1}\right) & ,y\;\left(\mathrm{mod}\;p_{1}\right))\times \ldots \times \left(x\;\left(\mathrm{mod}\;p_{k}\right),y\;\left(\mathrm{mod}\;p_{k}\right)\right), \end{array}\\ \begin{array}{cc} g(x & ,y)=\left(x\;\left(\mathrm{mod}\;p_{i}\right),y\;\left(\mathrm{mod}\;p_{i}\right)\right). \end{array} \end{array}$$

The MECs play an important role in cryptography. Azam et al. [58] defined different total orders, named as natural ordering (${≺}_{N}$) and diffusion ordering (${≺}_{D}$), on the points of MECs. For details, the readers are referred to [58].

## 3. The Proposed S-Box and Its Analysis

The proposed encryption scheme in Section 4 requires an S-box generator to obtain the desired level of confusion. For the construction of an S-box, we need the parameters n,b, and *t*. It is mentioned that $n=\prod_{i=1}^{k}p_{i}$, where each $p_{i}$ is a prime and *k* is a positive integer. In principle, *n* may be assigned any value. We choose *n* as a product of primes because, in the masking phase for each prime $p_{i}$, we need the EC over the related prime field $F_{p_{i}}$. The parameters n,b are used to generate the EC $E_{n,b}$ over the ring of integers $Z_{n}$, where *t* is used as an upper bound on the *y*-coordinate of the EC $E_{n,b}$. Here, upper bound *t* means that we compute such $\left(x,y\right)\in E_{n,b}$ for which y≤t. The *y*-coordinate is kept bounded, so that we compute $\left(x,y\right)\in E_{n,b}$ for all $x\in Z_{n}$ and particular values of *y* instead of all $y\in Z_{n}$. This is conducted in order to reduce the time of execution. After the generation of the EC $E_{n,b}$, the points of $E_{n,b}$ need to be arranged by some ordering. The points may be arranged according to any ordering, but the ordering ${≺}_{N}$ is capable of generating S-boxes with high cryptographic properties. Therefore, we use ${≺}_{N}$ to sort the points of $E_{n,b}$. Then, we construct an m×m S-box σ(n,t) by extracting the first $2^{m}$ different values $y_{i}<2^{m}$ of the *y*-coordinate of the ordered $E_{n,b}$. Mathematically, the S-box is generated according to the following function:

$$\sigma \left(n,t\right):\left[0,2^{m}-1\right]\to \left[0,2^{m}-1\right]$$

defined by:

$$\sigma \left(n,t\right)\left(i\right)=y_{i},$$

where $\left(x,y_{i}\right)\in E_{n,b}$ for some $x\in Z_{n}$ and $\sigma \left(n,t\right)\left(r\right)\neq y_{i}$ for r<i. The S-box construction is clearly described in the following steps: stepsenumerate1

Step 1. To generate an m×m S-box, select three integers n,b, and *t* such that $n>2^{m},0<b<n$ and $2^{m}\leq t\leq n$;
Step 2. Choose primes $p_{i}$ for a finite k≥2 in such a way that $n=\prod_{i=1}^{k}p_{i}$;
Step 3. For each y∈[0,t] and x∈[0,n−1], compute all the points (x,y) such that $y^{2}\equiv x^{3}+b\;\left(\mathrm{mod}\;n\right)$; i.e., compute $E_{n,b}$;
Step 1. If the set $\left[0,2^{m}-1\right]$ is contained in the *y*-coordinates of the points $\left(x,y\right)\in E_{n,b}$, then proceed to Step 5. Otherwise, change $p_{i}$ for some *i* and repeat Steps 1–3;
Step 5. Arrange the points of $E_{n,b}$ by applying the ordering ${≺}_{N}$;
Step 6. Construct the S-box $\sigma \left(n,t\right):\left[0,2^{m}-1\right]\to \left[0,2^{m}-1\right]$, such that $\sigma \left(n,t\right)\left(i\right)=y_{i}$, where $\left(x,y_{i}\right)\in E_{n,b}$ for some x∈[0,n−1] with the constraint that $y_{i}<2^{m}$ and σ(n,t)(j)≠σ(n,t)(i) for j<i.

For parameters $n=2491,p_{1}=47,p_{2}=53,b=716$, and t=255, an 8×8 S-box generated by the proposed method on an EC $E_{2491,716}$ is shown in Table 1.

### 3.1. Analysis of the Proposed S-Box

The cryptographic strength of the proposed S-box is analyzed by well-known tests, which are described as follows.

#### 3.1.1. Linear Attacks

A cryptosystem is secure if it can strongly resist attackers to exploit the linear relations of inputs and outputs. The immunity of an n×n S-box *S* against linear attacks is evaluated by its non-linearity NL(*S*) [59], linear approximation probability LAP(*S*) [60], and algebraic complexity AC(*S*) [61].

For a chosen *n*, the NL(*S*) represents the minimum Hamming distances between the S-box *S* and all the corresponding affine functions. Similarly, the LAP(*S*) is the approximation of the relation lying between the inputs and outputs, and the AC(*S*) represents the number of non-zero terms in the polynomial representation of the S-box *S*. Mathematically, the NL(*S*) and LAP(*S*) are computed by Equations (1) and (2), respectively:

(1) $$\begin{array}{c} \begin{array}{cc} \mathrm{NL}(S)= & \min_{\alpha \neq 0,\beta ,\lambda }\#\left\{x\in F_{2}^{n}:\alpha \cdot S\left(x\right)\neq \beta \cdot x⊕\lambda \right\}, \end{array} \end{array}$$

(2) $$\begin{array}{c} \begin{array}{cc} \mathrm{LAP}(S)= & \frac{1}{2^{n}}\left\{\max_{\alpha ,\beta \neq 0}\left\{|\#\left\{x\in F_{2}^{n}|x\cdot \alpha =S\left(x\right)\cdot \beta \right\}-2^{n-1}|\right\}\right\}, \end{array} \end{array}$$

where $\alpha ,\beta \in F_{2}^{n}$, and “· ”denotes the dot product. The resistance to linear attacks is greater if the NL(*S*) is close to $2^{n-1}-2^{(n/2)-1}$, the LAP(*S*) is low, and the AC(*S*) tends to $2^{n}-1$. The values of the NL, LAP, and AC for the S-box shown in Table 1 are 106,0.0156, and 254, respectively. Moreover, from Table 2, it follows that the NL of the proposed S-box is greater than those of [51,62,63,64,65,66] and equal to NL of the S-boxes in [8,12,37,48,58]. Similarly, the LAP of the new S-box is less than the LAP of the S-boxes in [8,12,37,48,51,58,62,63,64,65,66]. The AC of the presented S-box is greater than the AC of the S-boxes proposed in [51,58,64,65] and equal to that of the S-boxes [37,62,63,66]. From the above discussion, it is obvious that the proposed S-box is highly secure against linear attacks, compared to the S-boxes in [37,51,58,62,63,64,65,66], while the security of the proposed S-box against linear attacks is comparable with the S-boxes in [8,12,48].

#### 3.1.2. Differential Attacks

Differential attacks are used to study the differences of outputs for the corresponding differences of inputs to obtain useful information. For an S-box *S*, the differential approximation probability DAP(*S*) [67] measures the strength of *S* to thwart the differential attackers. The mathematical representation of DAP(S) is given by:

(3) $$\begin{array}{c} \mathrm{DAP}\left(S\right)=\frac{1}{2^{n}}\left\{\max_{\Delta x,\Delta y}\left\{\#\{x\in F_{2}^{n}|S\left(x⊕\Delta x\right)=S\left(x\right)⊕\Delta y\}\right\}\right\}, \end{array}$$

where $\Delta x,\Delta y\in F_{2}^{n}$, and “⊕” stands for the bit-wise addition in $F_{2}$. Thus, the S-box *S* possesses higher security if the DAP(*S*) is close to $1/2^{n}$. The DAP value of the S-box depicted in Table 1 is 0.0469. From Table 2, it is also evident that the DAP value of the proposed S-box is equal to the DAP values of the S-boxes displayed in [12,65,66], and comparable to the DAP of the S-boxes in [8,37,48,51,58,62,63,64]. Thus, the strength of the presented S-box against differential attacks is comparable to strength of the S-boxes in [8,12,37,48,51,58,62,63,64,65,66].

#### 3.1.3. Analysis of Boolean Functions

The Boolean functions of an S-box are used to create confusion/diffusion in a cryptosystem. Two approaches, strict avalanche criterion (SAC) [68] and bit independence criterion (BIC) [68], are used to analyze the Boolean functions. The SAC calculates the change that occurred in the output bits due to the inversion of one bit in a set of input bits. The BIC determines the dependence level of a pair of output bits on inverting an input bit. For an n×n S-box *S*, two matrices, $M\left(S\right)=\left[m_{ij}\right]$ and $B\left(S\right)=\left[b_{ij}\right]$, compute the SAC(*S*) and the BIC(*S*), respectively:

(4) $$\begin{array}{c} \begin{array}{c} m_{ij}=\frac{1}{2^{n}}\left(\sum_{x\in F_{2}^{n}}w\left(S_{i}{(x⊕\alpha }_{j}{)⊕S}_{i}\left(x\right)\right)\right), \end{array} \end{array}$$

(5) $$\begin{array}{c} \begin{array}{c} b_{ij}=\frac{1}{2^{n}}\left(\sum_{\begin{array}{c} x\in F_{2}^{n}\\ 1\leq r\neq i\leq n \end{array}}w\left(S_{i}\left(x⊕\alpha_{j}\right)⊕S_{i}\left(x\right)⊕S_{r}\left(x+\alpha_{j}\right)⊕S_{r}\left(x\right)\right)\right), \end{array} \end{array}$$

where w(y) represents the number of non-zero symbols in *y*, $\alpha_{j}\in F_{2}^{n}$ with $w(\alpha_{j})=1$, and $S_{i}$ denotes the *i*-th Boolean function of the S-box *S*. An S-box creates enough confusion/diffusion if all off-diagonal entries of M(S) and B(S) are close to 0.5. The minimum (SAC (min)) and maximum (SAC (max)) of the off-diagonal values of M(S) for the *S* displayed in Table 1 are 0.4063 and 0.5938, respectively. Furthermore, it follows from Table 2 that the SAC (min) value of the designed S-box is greater than the SAC (min) values of the S-boxes designed in [8,37,58], and that the SAC (max) value of the new S-box is less than or equal to that of the S-boxes in [8,12,37,58]. So, the confusion-creation capability of the S-box in Table 1 is greater than that of the S-boxes in [8,37,58]. The SAC (min) value of the presented S-box is equal to the SAC (min) values of the S-boxes in [12,62,65], and the SAC (max) value is less than that of the S-boxes in [12,62,65]. This reveals that the proposed scheme generates S-boxes with higher confusion than the schemes in [12,62,65]. The SAC values indicate that the confusion-creation capability of the new S-box is equal to the S-box in [51] and comparable to that of [48,66]. Now, the minimum (BIC (min)) and maximum (BIC (max)) of the off-diagonal values of B(S) for *S* in Table 1 are 0.4688 and 0.5293, respectively. Table 2 reveals that the BIC (min) of the proposed S-box is comparable with that of the S-boxes in [8,12,37,48,51,58,62,63,64,65,66], and the BIC (max) value of the current S-box is less than that of the S-boxes in [8,12,48,51,64,65,66]. Thus, the proposed scheme generates S-boxes with diffusion-creation capabilities comparable to the S-boxes in [8,12,37,48,51,58,62,63,64,65,66].

## 4. The Proposed Encryption Scheme

Suppose we want to encrypt an image *I* of size u×v over the symbol set $\left[0,2^{m}-1\right]$, and I(i,j) represents the pixel lying at the intersection of the *i*-th row and the *j*-th column. Furthermore, let $S_{I}$ denote the sum of all pixel values of *I*. Then, the proposed encryption scheme consists of the following steps.

### 4.1. Generation of Keys

To encrypt an image of size u×v, we need to generate an EC $E_{n,b}$, over a ring $Z_{n}$, for $n=\prod_{i=1}^{k}p_{i}$. For the sake of convenience, we take k=2 and n≥uv, so that we choose primes $p_{1},p_{2}$ and integers $b,t\in Z_{n}$ to generate $E_{n,b}$. Thus, $p_{1},p_{2},b$ and *t* are chosen in such a way that there exists at least uv points $\left(x,y\right)\in E_{n,b}$, where *y* is attaining each value in the interval $\left[0,2^{m}-1\right]$. This condition is imposed to ensure the generation of an S-box using points of the EC $E_{n,b}$. We further choose an integer value $\ell_{1}$ as a key. The use of the key $\ell_{1}$ is explained in Section 4.4(i). The parameters $p_{1},p_{2},b,t$, and $\ell_{1}$ are all secret keys.

### 4.2. Masking Phase

Before masking an image *I*, we first arrange the points of the EC $E_{n,b}$ according to the ordering ${≺}_{D}$. After ordering, we assume that $\left(x_{i},y_{i}\right)\in E_{n,b}$ stands for the *i*-th point of the EC $E_{n,b}$. The reason for this is that the ${≺}_{D}$ diffuses the *y*-coordinates of the points lying on an EC, because, over a ring of integers $Z_{n}$, if $\left(x,y\right)\in E_{n,b}$, then, for such an *x*, there are at least two values $y_{1},y_{2}$ of *y* in $Z_{n}$, such that $\left(x,y_{1}\right),\left(x,y_{2}\right)\in E_{n,b}$. The masking phase of an image *I* takes place as follows:

(i) Generate a row matrix *M* from $\left(x_{i},y_{i}\right)\in E_{n,b}$ of length $2(\#E_{n,b})$ such that, for $1\leq i\leq \#E_{n,b}$, we have:
  $$M\left(j\right)=\left\{\begin{array}{cc} x_{i}, & \mathrm{if}\;j\;\mathrm{is}\;\mathrm{odd}\\ x_{i}+y_{i}, & \mathrm{otherwise} \end{array}\right.$$
  The purpose of constructing matrix *M* is to design a sequence from both coordinates of $E_{n,b}$. The elements of the said sequence are used to hide the pixel values of an image *I*;
(ii) Choose a submatrix *N*, which consists of the first uv entries of the matrix *M* because only the uv values are needed to hide the pixel values. The chosen *n* should not be less than uv; otherwise, in Section 4.3(i), the construction of $M_{2}$ with size u×v will not be possible;
(iii) The sensitivity to the plain image is necessary for a secure cryptosystem. For this purpose, transform the entries of *N* by the pixel value I(1,1) to obtain the matrix *B*, given by:
  (6) B(i)=N(i)+I(1,1).
  There is no restrictions on pixel or the number of pixels. Any number of arbitrary pixels may be used for transformation. For the sake of convenience, only one pixel value I(1,1) is fixed;
(iv) For the sake of simplicity, reshape the matrix *B* to construct a matrix with *u* rows and *v* columns. By reshaping *B*, we mean that *B* is divided into *v* blocks such that each block contains *u* entries and the *i*-th block represents the *i*-th column of the matrix *B*, so that the corresponding values of both *B* and *I* are combined to hide the pixel values of the image *I*;
(v) To obtain the masked image $M_{I}$, mask the pixels of image *I* using Equation (7):
  (7) $$M_{I}\left(i,j\right)=I\left(i,j\right)+B\left(i,j\right)\;\left(\mathrm{mod}\;2^{m}\right).$$

Since there is a one–one correspondence between $E_{n,b}$ and $E_{p_{1},b}\times E_{p_{2},b}$, $E_{n,b}$ may be mapped onto $E_{p_{1},b}$ and $E_{p_{2},b}$, respectively, via the following maps:

(8) $$\begin{array}{c} \left(x,y\right)\to (x\;\left(\mathrm{mod}\;p_{1}\right),\;y\;\left(\mathrm{mod}\;p_{1}\right)), \end{array}$$

(9) $$\begin{array}{c} \left(x,y\right)\to (x\;\left(\mathrm{mod}\;p_{2}\right),\;y\;\left(\mathrm{mod}\;p_{2}\right)). \end{array}$$

These two ECs, $E_{p_{1},b}$ and $E_{p_{2},b}$, are used to alter the pixels of an image. The S-box generated on $E_{n,b}$ is used to create the confusion in the encrypted image. The steps of the said procedure are explained in the following phase.

### 4.3. Diffusion Phase

The steps of this phase are explained as follows:

(i) For $1\leq i\leq \#E_{n,b}$, construct two row matrices, $M_{k},k=1,2$, due to the all points $\left(x_{ik},y_{ik}\right)\in E_{p_{k},b}$ for both primes $p_{k},k=1,2$, respectively, such that:
  $$M_{1}\left(j\right)=\left\{\begin{array}{cc} x_{i1}, & \mathrm{if}\;j\;\mathrm{is}\;\mathrm{odd}\\ x_{i1}+y_{i1}, & \mathrm{otherwise}, \end{array}\right.$$
  and:
  $$M_{2}\left(j\right)=y_{i2}.$$
  In fact, we want to generate two sequences using ECs $E_{p_{1},b}$ and $E_{p_{2},b}$. Both sequences consist of integer values, which are further used to alter the pixel values of the masked image $M_{I}$ in Section 4.3(v) and to permute the image *P* in Section 4.4(iii), respectively;
(ii) Take submatrices $N_{k},k=1,2$ containing the first uv entries of $M_{k},k=1,2$, respectively, so as to choose the sequences of length that are equal to that of the number of pixels in the image *I*;
(iii) Modify the sizes of above constructed matrices, so that $N_{k},k=1,2$ has *u* rows and *v* columns. The reason for generating such matrices has been explained previously;
(iv) Apply the modulo $2^{m}$ operator to $N_{k},k=1,2$ to generate the matrices $B_{k},k=1,2$, consisting of *m*-bit integers. Since for encrypting *m*-bit images, *m*-bit sequences are needed;
(v) Convert the elements of $M_{I}$ and $B_{k},k=1,2$ into the binary format and generate the image $X_{1}$ by diffusing the pixels of $M_{I}$ by the following equation:
  (10) $$X_{1}\left(i,j\right)=M_{I}⊕B_{1}\left(i,j\right),$$
  where ⊕ is a logical operator (XOR operation by binary bit) known as exclusive disjunction.

### 4.4. Confusion Phase

For a secure cryptographic algorithm, it is necessary to have a desired level of confusion. For the current cryptosystem, the confusion phase consists of the following steps:

(i) Choose a secret key $\ell_{1}$ to construct a shifting parameter $\ell_{2}$, such that $\ell_{2}=S_{I}+\ell_{1}\;\left(\mathrm{mod}\;2^{m}\right)$; then, give a circular shift to the S-box σ(n,t) to design a new S-box $\sigma (n,t,\ell_{2})$. The shifting parameter, the secret key $\ell_{1}$, and $S_{I}$ are linked in order to enhance the sensitivity to the plain image *I*;
(ii) Permute the pixels of the image $X_{1}$ using the S-box $\sigma (n,t,\ell_{2})$ as follows:
  (11) $$P\left(i,j\right)=\sigma \left(n,t,\ell_{2}\right)X_{1}\left(i,j\right).$$
  In the coming encryption of a 4×4 hypothetical image, the first entry of $X_{1}$ is 2. Then, $\sigma \left(n,t,\ell_{2}\right)\left(2\right)$ represents the third entry of the S-box $\sigma (n,t,\ell_{2})$, which is 1. That is, $\sigma \left(n,t,\ell_{2}\right)\left(r\right)$ stands for the (r+1)-th element of the S-box $\sigma (n,t,\ell_{2})$;
(iii) In order to obtain the scrambled image $X_{2}$, repeat Section 4.3(v) using *P* in place of $M_{I}$, and replacing $B_{1}\left(i,j\right)$ with $B_{2}\left(i,j\right)$, such that:
  (12) $$X_{2}\left(i,j\right)=P⊕B_{2}\left(i,j\right);$$
(iv) Finally, to obtain the encrypted image *C* with the desired level of confusion, permute the image $X_{2}$ as follows:
  (13) $$C\left(i,j\right)=\sigma \left(n,t,\ell_{2}\right)X_{2}\left(i,j\right).$$

**Figure 1:**
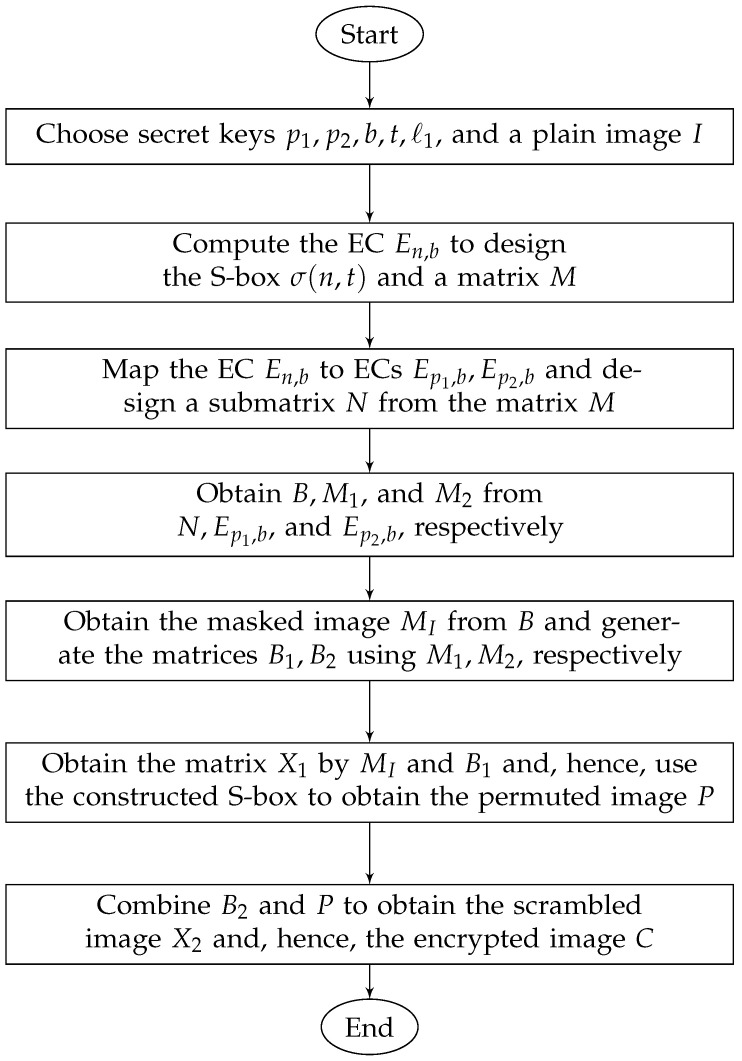
Flowchart of the encryption scheme.

The flowchart of the proposed encryption scheme is shown in Figure 1.

We theoretically derive the time complexity of the proposed scheme in Theorem 1.

**Theorem** **1.** *Let I be a plain image of size u×v, and let n≥uv be a positive integer; then, the time complexity of the proposed scheme is $O\left(\max\{nt,\left(\#E_{n,b}\right)\log\left(\#E_{n,b}\right)\}\right)$, where t≤n is an integer such that $E_{n,b}$ is computed for all x∈[0,n−1] and y≤t.*

**Proof.**  In the key generation phase (Section 4.1), to compute all $\left(x,y\right)\in E_{n,b}$, we need to check for each x∈[0,n−1], *t* values of y∈[0,t−1], such that $y^{2}\equiv x^{3}+b\;\left(\mathrm{mod}\;n\right)$. Thus, the computation of $E_{n,b}$ takes O(nt) time. Further, the ordering of the points of the EC $E_{n,b}$ needs $O\left(\left(\#E_{n,b}\right)\log\left(\#E_{n,b}\right)\right)$ time. In Section 4.2(i), we can see that $\#M=2(\#E_{n,b})$; therefore, *M* can be constructed in O(n) time. As #N=#B=uv, we can design N,B by a for loop, executing uv times. Furthermore, *B* can be reshaped by two nested loops, such that one loop executes *u* times, while the other executes *v* times. In a similar way, we can add two matrices of the same order by two nested loops. Thus, the time complexity of Section 4.2(ii)–(v) is O(uv). From the chosen keys, we have n≥uv and $\#E_{n,b}\leq n$, so that the complexity of the masking phase is $O\left(\max\{nt,\left(\#E_{n,b}\right)\log\left(\#E_{n,b}\right)\}\right).$

Now, we can map the EC $E_{n,b}$ on the ECs $E_{p_{1},b},E_{p_{2},b}$ in O(n) time. In the diffusion phase (Section 4.3(i)), $\#M_{k}=2\left(\#E_{n,b}\right)$ and $\#N_{k}=\#B_{k}=uv$ for k=1,2. Therefore, Section 4.3(i)–(v) takes $O(\#E_{n,b})$ and O(uv) time, respectively.

In the confusion phase (Section 4.4(i)), the time required to compute $S_{I}$ is O(uv) and $\ell_{2}$ can be computed in constant time. Since $X_{1}$ has uv entries, we can implement Section 4.4(ii)–(iv) in O(uv) time, using two nested loops executing *u* and *v* times, respectively.

Clearly, nt>n and n≥uv; thus, the above discussion implies that the time complexity for the presented scheme is $O\left(\max\{nt,\left(\#E_{n,b}\right)\log\left(\#E_{n,b}\right)\}\right)$. □

As the time complexity is dependent on the parameter *t*, that is, the time is controllable with *t*, the current scheme is effective, particularly when a large dataset of images is to be encrypted.

**Figure 2:**
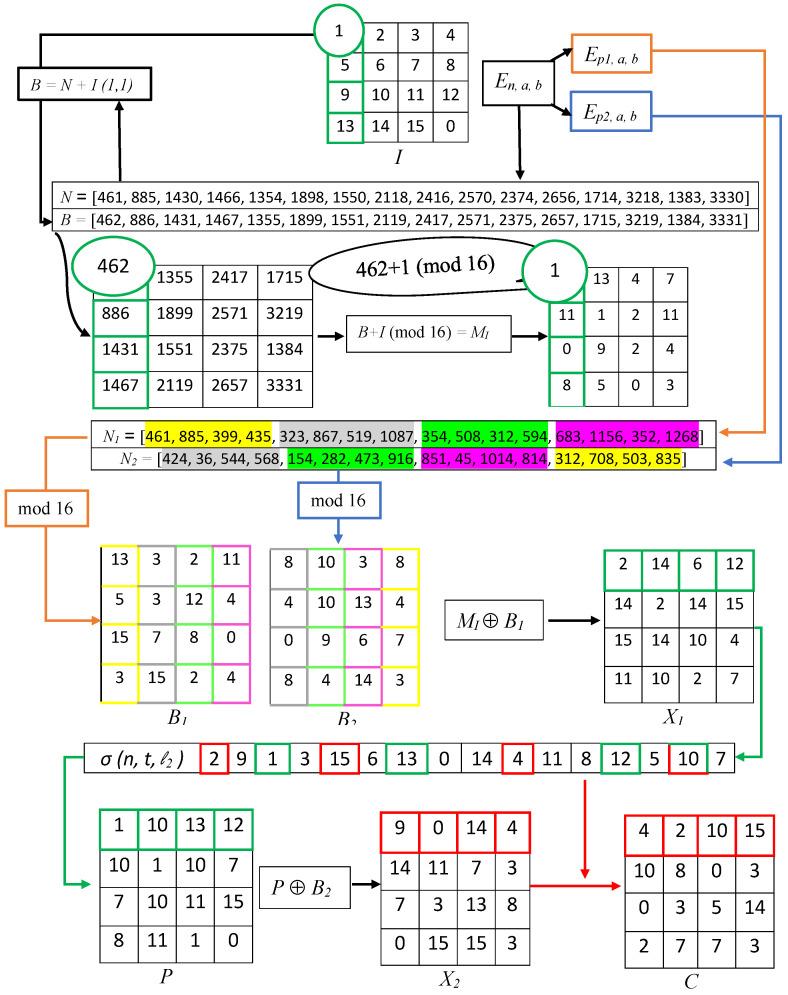
Encryption of a 4×4 image by the proposed scheme.

The whole process is illustrated by the encryption of a 4-bit hypothetical image, as shown in Figure 2.

## 5. Decryption

The decryption process takes place by reversing all the steps of the encryption process. The receiver will need the keys $p_{1},p_{2},b,t$, and $\ell_{1}$ to generate the inverse S-box $\sigma^{-1}\left(n,t,\ell_{2}\right)$ and the matrices $B_{k},k=1,2$. Then, by the use of Equations (6), (7), and (10)–(13), one can obtain the original image *I*.

## 6. Security Analysis

**Figure 3:**
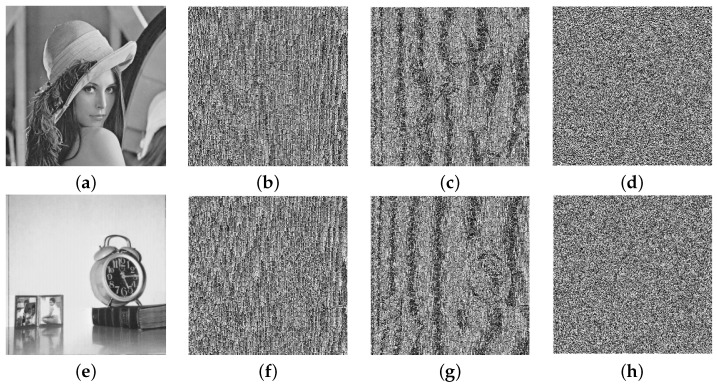
(**a**,**e**) Plain images; (**b**,**f**) masked images; (**c**,**g**) diffused images; (**d**,**h**) encrypted images of Lena and Clock, respectively.

In this section, some well-known metrics are described, which are generally used to measure the security level of new algorithms. The security of the proposed encryption scheme is evaluated by performing experiments on all-gray images of different sizes, taken from the USC-SIPI database [69]. The database consists of square images of size w×w, w=256,512,1024. The plain images of Lena and Clock, along with their encrypted images, are shown in Figure 3. The experiments are performed by taking the parameters $p_{1}=p_{2}=1031,b=7,t=1031^{2}$ and $\ell_{1}=80-S_{I}$, using MATLAB 2016a on a personnel machine equipped with a 1.8 GHz processor, 6 GB RAM, and Windows 10 operating system. The security strength of the encryption algorithm is analyzed in the following subsections.

### 6.1. Differential Attacks Analysis

**Figure 4:**
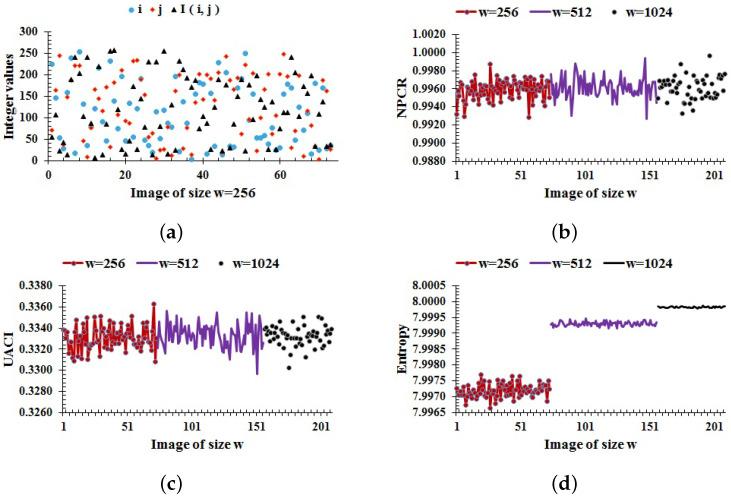
(**a**) Random values of i,j and I(i,j); (**b**–**d**) NPCR, UACI, and entropy results, respectively, for the whole image database.

The cryptographic strength of an encryption scheme against differential attacks is analyzed by two measures: the number of pixels’ change rate (NPCR) and the unified average changing intensity (UACI). In an encryption algorithm, the change of one pixel of a plain image has an influence on the encrypted image. The NPCR is a criterion used to measure the influence of a change in a plain image on the ciphers. The UACI criterion measures the average intensity difference between two different images. If *I* and $I^{\prime }$ are any two plain images of the same dimension u×v, and $C_{I}$ and $C_{I^{{}^{\prime }}}$ are the respective cipher images of *I* and $I^{\prime }$, then NPCR and UACI are calculated by Equations (14) and (15), as given by:

(14) $$\begin{array}{cc} \mathrm{NPCR} & =\sum_{i=1}^{u}\sum_{j=1}^{v}\frac{\tau (i,j)}{u\times v}, \end{array}$$

(15) $$\begin{array}{cc} \mathrm{UACI} & =\sum_{i=1}^{u}\sum_{j=1}^{v}\frac{|C_{I}\left(i,j\right)-C_{I^{{}^{\prime }}}\left(i,j\right)|}{255\times u\times v}, \end{array}$$

where τ(i,j)=0 if $C_{I}\left(i,j\right)=C_{I^{{}^{\prime }}}\left(i,j\right)$, and τ(i,j)=1, otherwise. For 8-bit images with w=256,512,1024, the theoretical values of NPCR are 99.5693,99.5893,99.5994, respectively. Unlike NPCR, the expected ranges of UACI are [33.2824,33.6447], [33.3730, 33.5541], and [33.4183,33.5088], respectively [18]. We randomly choose *i* and *j*, and a random value is assigned to the pixel I(i,j) for each image *I* of the database. The random values of i,j and I(i,j) for images of size w=256 are shown in Figure 4a. Then, the NPCR and UACI results for the current scheme are computed for each image of the database for a randomly chosen pixel value I(i,j); the graphical results are shown in Figure 4b,c, where the average values of NPCR and UACI are 99.60 and 33.32, respectively, which are close enough to the expected values.

Moreover, we randomly changed one pixel value of some images, and compared the results of the NPCR and UACI tests with the results of the recent schemes in [14,15,16,17,18,28,51,57,70,71], as shown in Table 3 and Table 4, respectively. The pass ratio for the proposed scheme in Table 3 is higher than that of the schemes in [14,15,17,28,51], and equal to that of the schemes in [16,18,57,70,71]. Similarly, the pass ratio for the proposed scheme in Table 4 is higher than that of the schemes in [15,16,17,51,57,70] and equal to that of the schemes in [14,18,28,71]. It follows that the proposed scheme has better performance against differential attacks than the schemes in [14,15,16,17,28,51,57], and has comparable performance to the schemes in [18,71].

### 6.2. Information Entropy

Information entropy is used to measure disorder in an image. Equation (16) is used to determine the randomness of an image *I*:

(16) $$H\left(I\right)=-\sum_{i=1}^{k}p\left(x_{i}\right)\log_{2}\left(p\left(x_{i}\right)\right),$$

where $p(x_{i})$ represents the probability of a pixel value $x_{i}$, and *k* is the total number of gray values in an image *I*. For an 8-bit encrypted image, the ideal value of entropy is 8, which corresponds to the highest level of uncertainity. Thus, for a cryptographically strong encryption scheme, the value of H(I) should be close to 8. The entropy results for the current scheme are computed for each image of the database as shown in Figure 4d. The entropy for images of size w=256,512,1024 are lying in the ranges [7.9966,7.9977], [7.9991,7.9995], and [7.9998,7.99987], respectively. In all cases, the entropy approaches the optimal value. Consequently, the proposed encryption scheme is capable of providing high randomness in a cipher.

The comparison of the entropy results is carried out in Table 5. It is clear from Table 5 that the information entropy for the presented scheme is higher than that of [15,16,17,19,20,28,46,51,57] and comparable to that of the schemes in [14,18,41,72]. Thus, our scheme generates encrypted images having more randomness than the techniques in [15,16,17,19,20,28,46,51,57.

### 6.3. Histogram

**Figure 5:**
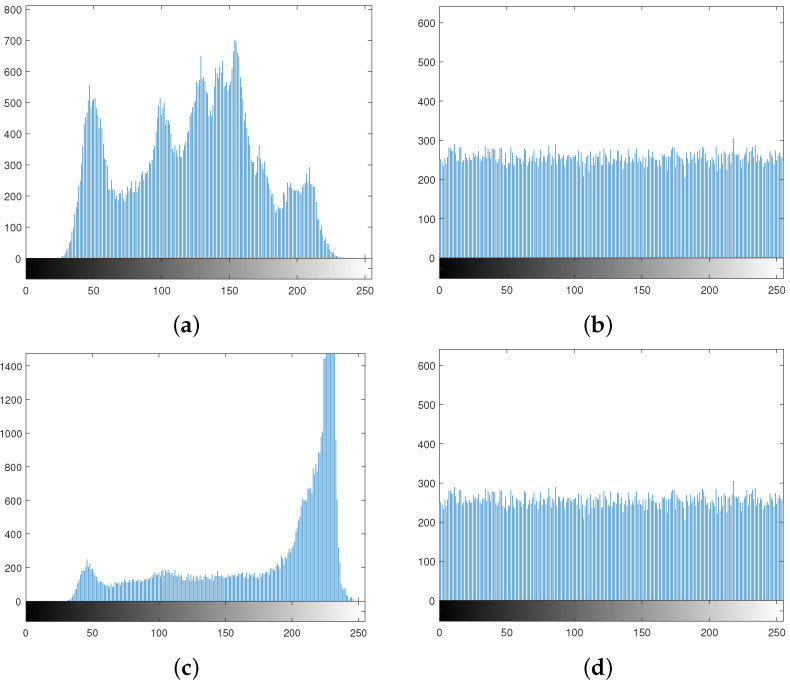
Histograms of plain and encrypted images: (**a**,**c**) histogram of the plain images in Figure 3a,e, respectively; (**b**,**d**) histogram of the encrypted images in Figure 3d,h, respectively.

A histogram of an image represents the frequency distribution of the gray values. If each pixel value occurs with almost equal frequency in an image, then the histogram of that image is said to be uniform. The histogram of an ordinary image is always highly nonuniform, while a properly encrypted image has a uniform histogram. Figure 5a,c depicts the histograms of the plain images in Figure 3a,e, repectively, and Figure 5b,d shows the histograms of the cipher images in Figure 3d,h, respectively.

It is evident from the histograms that the proposed scheme encrypts an image in such a way that it does not reveal any secret information of the former.

### 6.4. Correlation

**Figure 6:**
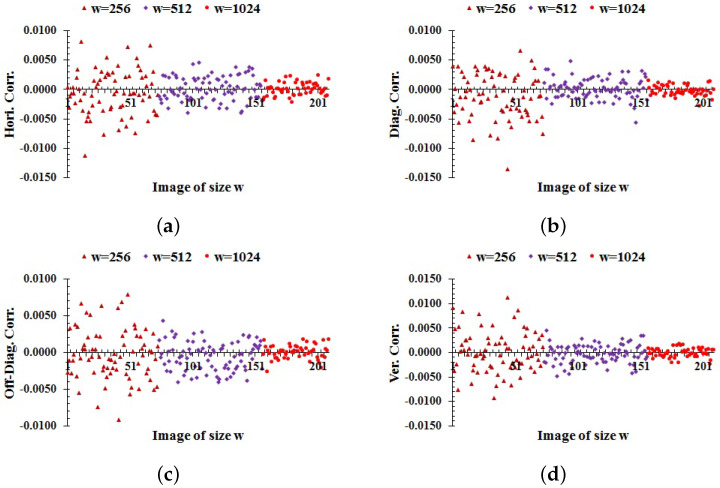
Correlation among the adjacent pixels of each encrypted image in the databases: (**a**) horizontal; (**b**) diagonal; (**c**) off-diagonal; (**d**) vertical correlation.

A pixel of an ordinary image has high correlation with adjacent pixels. A good encryption scheme breaks the correlation among the pixels of an encrypted image. The correlation coefficient between datasets *x* and *y* of the same size *M* is determined by:

(17) $$C_{xy}=\frac{\sum_{i=1}^{M}\left(x_{i}-E\left[x\right]\right)\left(y_{i}-E\left[y\right]\right)}{\sqrt{\sum_{i=1}^{M}{\left(x_{i}-E\left[x\right]\right)}^{2}\sum_{i=1}^{M}{\left(y_{i}-E\left[y\right]\right)}^{2}}},$$

where $x_{i}\in x$ and $y_{i}\in y$ and $E\left[x\right]=\frac{1}{M}\sum_{i=1}^{M}x_{i}$. The horizontal, diagonal, off-diagonal, and vertical correlation coefficients among the pixels of each image in the database encrypted by the proposed scheme are computed as shown in Figure 6. The average values of correlation in Figure 6a–d are −0.00012,−0.00038,−0.00026, and −0.00004, respectively. From these results, it follows that the proposed scheme makes the correlation close to zero in all directions. So, the proposed method is capable of disrupting the correlation of pixels in ciphers.

**Figure 7:**
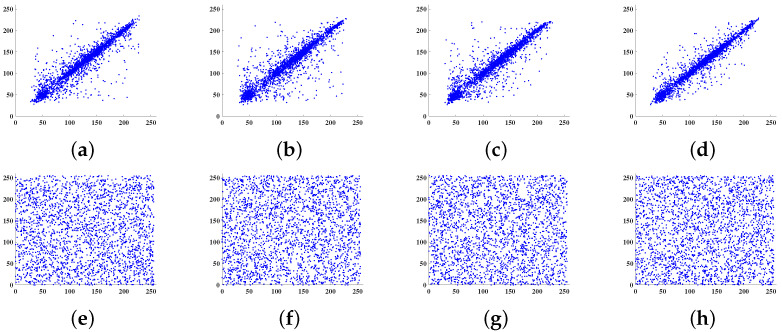
Correlation distribution of adjacent pixels of plain image along the (**a**) horizontal, (**b**) diagonal, (**c**) off-diagonal, and (**d**) vertical directions, respectively; correlation distribution of adjacent pixels of cipher image along the (**e**) horizontal, (**f**) diagonal, (**g**) off-diagonal, and (**h**) vertical directions, respectively.

In addition, a random sample of 2560 pairs of pixels is selected along the horizontal, diagonal, off-diagonal, and vertical directions from both the plain image and the cipher image of Lena${}_{512\times 512}$. The correlation distribution between the adjacent gray values before and after encryption is shown in Figure 7.

It follows From Figure 7a–d that adjacent pixels of the plain image are in high correlation, but Figure 7e–h indicates that the proposed scheme successfully weakens the correlation of the pixels.

The correlation results for encrypted image of Lena${}_{512\times 512}$ and Barbara${}_{512\times 512}$ are compared with other schemes in Table 6. It can be observed that the results for the Lena image of the current scheme are better than all the compared schemes. Similarly, the results for the Barbara image encrypted by the proposed scheme are better than that of the schemes in [19,41,51,72], and comparable to the results of [16,57]. Thus, our scheme generates encrypted images with more randomness than the other techniques listed in Table 6.

### 6.5. Key Space

Key space is a set consisting of the all possible secret keys for a cryptosystem. Generally, for a good cryptosystem, the size of a key space should be at least $2^{128}.$ The five keys $p_{1},p_{2},b,t$, and $\ell_{1}$ are introduced by the proposed scheme. The least number of bits to store a key required by the proposed algorithm is 29. Thus, the size of our key space is $2^{145}$, which is much larger than $2^{128}$. Hence, the described scheme has the capability to resist brute-force attacks in an efficient way.

### 6.6. Key Sensitivity

**Figure 8:**
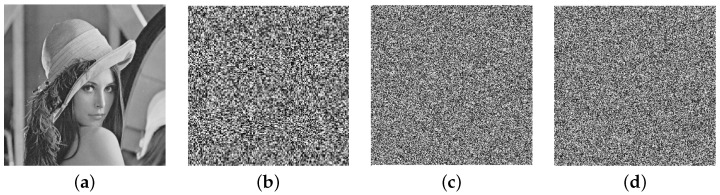
Decrypted image with (**a**) actual keys; (**b**) $p_{1}=p_{2}=257$; (**c**) b = 8; (**d**) $\ell_{1}=\ell_{1}+1$.

This is an important feature of a cryptographically strong cryptosystem. Key sensitivity is also necessary to resist brute-force attacks. If a small change in a key leads to a significant change in the cipher, then the cryptosystem is said to be sensitive to the keys. For this purpose, we decrypted the Lena image by changing a single key; the results are shown in Figure 8b–d.

**Figure 9:**
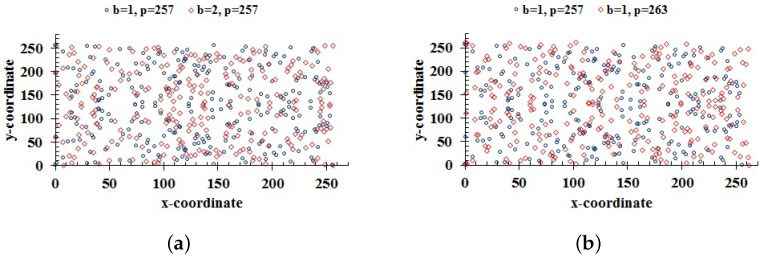
(**a**) Two ECs generated for a small change in the key *b*; (**b**) points of ECs for two different primes.

Moreover, slightly changing the keys *b* and *p* change the coordinates of the ECs $E_{257,1},E_{257,2}$, and $E_{257,1},E_{263,1}$; these are shown in Figure 9a,b, respectively.

Figure 9 shows the sensitivity of the masking phase to the parameters of the ECs. From Figure 8 and Figure 9, it follows that slight changes in a key lead to very different results. Hence, our proposed scheme is highly sensitive to the keys.

### 6.7. Plain-Text Attacks

**Figure 10:**
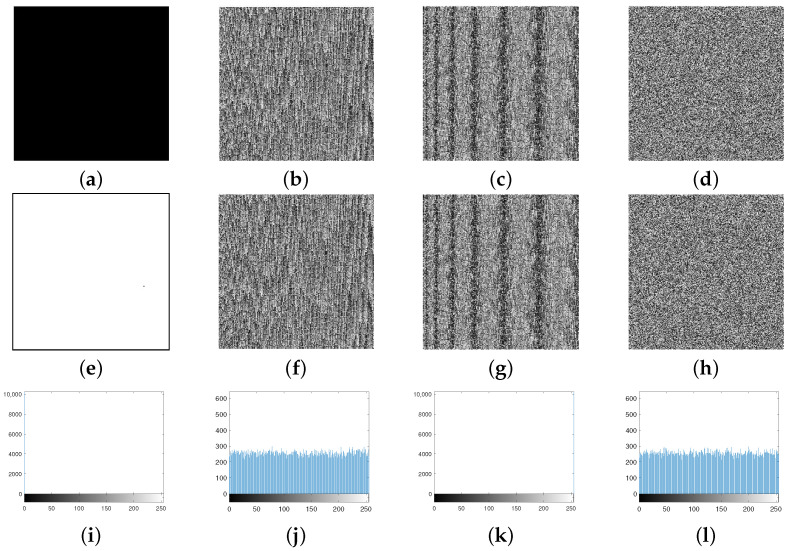
(**a**,**e**) Plain images; (**b**,**f**) masked images; (**c**,**g**) diffused images; (**d**,**h**) encrypted images of all-black and all-white images, respectively; (**i**,**k**) histograms of (**a**,**e**), respectively; (**j**,**l**) histograms of (**d**,**h**), respectively.

There are two types of plain-text attacks, i.e., known plain-text attacks and chosen plain-text attacks. In known plain-text attacks, the attacker knows about a string of the plain text, along with the relevant string of the cipher text. In chosen plain-text attacks, the adversary has a partial access to the encryption scheme. That is, the adversary can obtain the ciphered string for a chosen plain-text string. For this purpose, attackers use all-black or all-white images to obtain information about the encryption scheme [50], so that a secure encryption scheme encrypts all-black and all-white images with optimal results. The efficiency of the current scheme is visible from Table 7 and Figure 10.

Table 7 and Figure 10 indicate that the current scheme not only randomizes the all-white and all-black images, but also weakens the pixels’ correlation and makes the pixels’ distribution uniform in the encrypted images. Thus, the proposed scheme is highly secure against both kinds of plain-text attacks.

## 7. An Application to Encryption of Color Images

**Figure 11:**
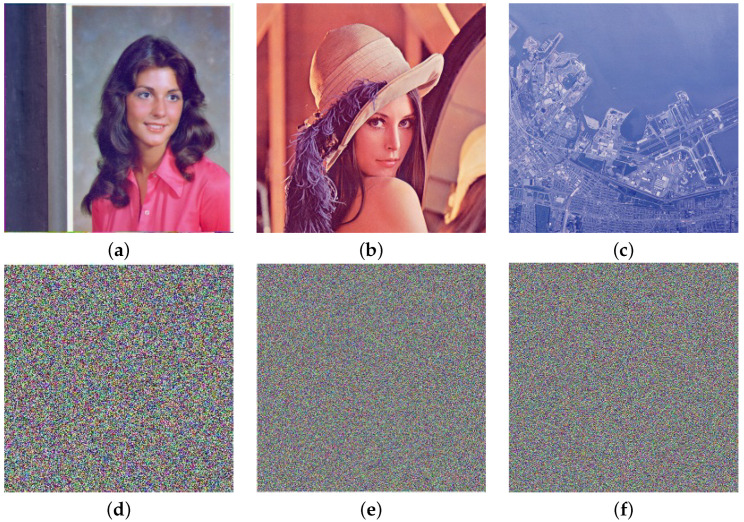
(**a**–**c**) Plain images; (**d**–**f**) encrypted images of Female${}_{256\times 256}$, Lena${}_{512\times 512}$, and San Francisco${}_{1024\times 1024}$, respectively.

In this section, we apply the proposed scheme to the color images. We encrypted images of Female${}_{256\times 256}$, Lena${}_{512\times 512}$, and San Francisco${}_{1024\times 1024}$ using the parameters $p_{1}=p_{2}=1031,b=7,t=1031^{2}$, and $\ell_{1}=80-S_{I}$, where *I* stands for the R (Red), G (Green), and B (Blue) components of an image, respectively. The plain and encrypted images of Female${}_{256\times 256}$, Lena${}_{512\times 512}$, and San Francisco${}_{1024\times 1024}$ are shown in Figure 11.

The NPCR and UACI results are computed by randomly changing a pixel value in each channel. It can be observed from Table 8 that the NPCR and UACI results of each channel for the tested color images lie in the expected range. Similarly, the entropy results of the R, G, and B components also belong to the expected ranges for each of the color images.

The experimental results of the color Lena${}_{512\times 512}$ are compared with the results of the schemes in [7,28,29,73,74,75,76,77,78], as shown in Table 9 and Table 10.

The results in Table 9 reveal that the NPCR values for the R, G, and B components of Lena${}_{512\times 512}$ are greater than the theoretical value (99.5893). Apart from this, the R component’s NPCR value is greater than the values of the schemes in [29,75], equal to that of [7,77], and comparable with [28,74,76]. The NPCR value of the G plane is greater than all the NPCR results of to the schemes in [7,28,29,73,74,75,76,77]. Similarly, the NPCR results of the B component is better than the results of [7,28,29,74,75,76,77,78]. In addition, the average NPCR value resulting from the current scheme is greater than the NPCR results of all the listed schemes in Table 9. The UACI results of the R, G, and B components of the current scheme lie in the expected range. Furthermore, the UACI results of the new scheme for all the three components are better than the results of the schemes in [7,73,75,78] and comparable to that of the schemes in [28,29,74,76,77]. It follows that the proposed scheme is better in performance against the differential attacks than the schemes in [7,28,70,73,74,75,76,77,78].

Table 10 reveals that the entropy value of the R component is greater than that in [74,75,77] and equal to the values of [76,78]. The entropy value of the G component is also greater than that in [74,75,77] and equal to the value of [29]. Similarly, the entropy result of the B component is greater than the results of [28,29,43,73,74,75,77,78] and equal to that of [7,76]. In addition, the average entropy value resulting from the current scheme is better than that of the schemes in [73,74,75,77] and equal to that of the results of [28,29,43,76,78]. This discussion indicates that the current scheme generates encrypted color images with higher randomness than the schemes in [74,75,77], and the randomness of the encrypted images in current scheme and the schemes in [29,78] is comparable.

**Figure 12:**
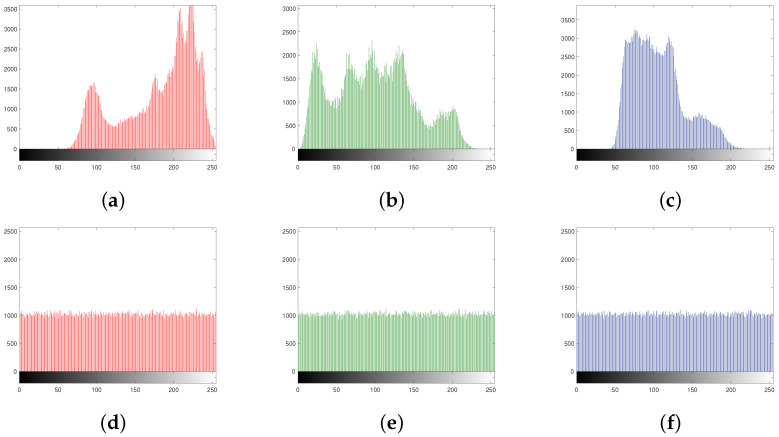
(**a**–**c**) Histogram of the plain R, G, and B channels of the color Lena${}_{512\times 512}$, respectively; (**d**–**f**) histogram of the encrypted R, G, and B channels of the color Lena${}_{512\times 512}$, respectively.

The histograms of the channels of the plain Lena${}_{512\times 512}$ and the encrypted Lena${}_{512\times 512}$ are shown in Figure 12a–f, respectively.

Figure 12 confirms that the histograms of the encrypted channels are uniform and, hence, the presented scheme encrypts color images having high resistance against the statistical attacks.

In Table 11, the correlation of the adjacent pixels of three different encrypted images with different sizes is shown. It is evident that the proposed scheme encrypts any image in such a way that it weakens the correlation between two adjacent pixels of any channel.

**Figure 13:**
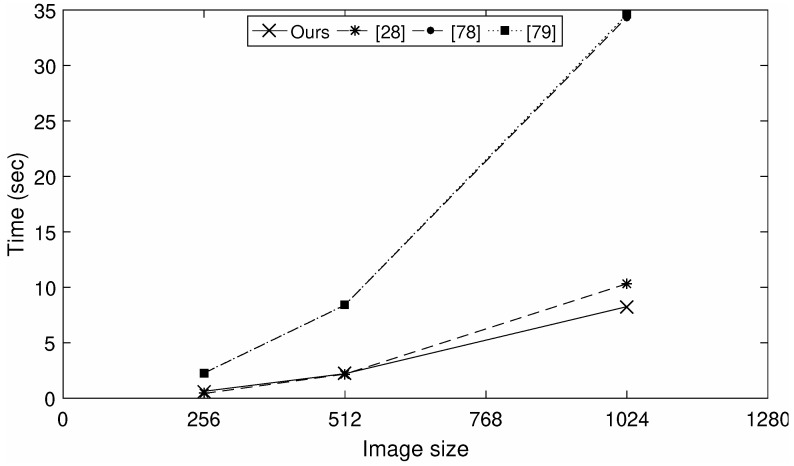
Encryption time for color images according to different encryption schemes.

Along with other properties, a good encryption scheme should be highly efficient. Different color images with different sizes are encrypted using the current scheme. To demonstrate the efficiency of the current scheme, the encryption times (sec) for the said three images are computed, since we use a pre-computed EC over the ring of integers as an input for all input images. While computing the encryption time, the time taken by the inputs is not taken under the consideration. The encryption time of the current scheme is compared with the time of some recent schemes [28,78,79], as shown in Figure 13. The results for the schemes in [28,78,79] are available in Table 3 of [80].

For images of size *w* = 256,512, the performance of our scheme is comparable with that of [28], and for w=1024, the new scheme is highly efficient, compared to the scheme of [28]. Similarly, in Figure 13, the plots of [78,79] are overlapping, but our scheme is more efficient than [78,79] for images of all sizes.

Thus, the presented scheme is capable of efficiently encrypting color images as well, and can be used for the good encryption of color images.

## 8. Conclusions

We proposed a new S-box generator and an image encryption algorithm. We employed an EC over a ring of integers instead of a finite field. The presented S-box is highly resistive against linear attacks when compared to the S-boxes generated by the ECs over finite fields in [37,51,58] and the S-boxes of [62,63,64,65,66]. The confusion-creation capability of the proposed S-box is higher than that of the S-boxes in [8,37,58].

Furthermore, our encryption scheme has better performance against differential attacks than that of the EC-based schemes [51,57] and the schemes in [14,15,16,17,28], and provides encrypted images with higher randomness than the schemes in [15,16,17,19,20,28,51,57]. The current scheme is also used for different color images. The presented scheme is able to encrypt color images with low run-times and higher security when compared with [78,79], while the scheme in [51] discusses the novelty regarding only gray images. Furthermore, for relatively large images, the run-time of the current scheme is very low, compared to the schemes in [28,78,79]. The future directions consist of the following works:

(i) To improve the current scheme for the simultaneous encryption of all channels of a color image; (ii) To optimize the current scheme for a text-encryption algorithm; (iii) To generate random numbers based on ECs over rings and employ the sequence of random numbers in text encryption; (iv) To generate random binary sequences using ECs over a ring of integers and experimentally prove their cryptographic strength.

## Figures and Tables

**Table 1:** The S-box σ(2491,255) generated by the proposed algorithm.

29	221	121	55	244	223	215	53	14	115	131	96	11	143	145	238
136	3	181	138	137	248	61	189	140	64	20	182	70	134	124	49
141	50	19	191	164	125	22	179	118	237	202	66	10	167	38	83
40	193	230	234	175	9	159	158	229	34	251	205	249	180	197	81
218	110	195	116	27	150	94	87	157	62	48	226	117	75	217	99
86	1	68	90	165	130	242	163	32	203	72	51	89	235	37	120
113	77	239	188	5	201	162	60	149	192	211	59	161	88	155	6
233	222	106	8	178	245	209	123	204	199	76	153	122	194	184	100
246	228	107	142	26	207	232	104	227	198	73	154	186	172	152	12
57	56	65	58	44	69	46	74	92	101	174	160	82	220	112	200
135	177	148	147	213	24	208	129	168	35	169	30	42	144	216	127
241	255	33	95	171	7	109	170	28	247	212	98	225	210	84	31
93	47	219	119	176	108	156	240	166	196	52	36	114	190	67	173
25	71	15	139	128	39	252	151	17	105	78	80	214	254	16	111
43	13	250	85	79	183	45	21	0	146	126	102	63	187	185	132

**Table 2:** Comparison of the S-boxes generated by the proposed algorithm and some recent algorithms.

Scheme	NL	LAP	AC	DAP	SAC (min)	SAC (max)	BIC (min)	BIC (max)
Proposed	106	0.0156	254	0.0469	0.4063	0.5938	0.4688	0.5293
Ref. [37]	106	0.1484	254	0.0234	0.3906	0.6094	0.4727	0.5254
Ref. [58]	106	0.1328	253	0.0391	0.3750	0.5938	0.4688	0.5254
Ref. [62]	104	0.0469	254	0.0391	0.4063	0.6250	0.4668	0.5234
Ref. [63]	101	0.0664	254	0.0391	0.4219	0.5781	0.4668	0.5195
Ref. [64]	104	0.0625	253	0.0391	0.4219	0.5938	0.4766	0.5391
Ref. [65]	100	0.0391	253	0.0469	0.4063	0.6094	0.4473	0.5332
Ref. [8]	106	0.0703	255	0.0391	0.3906	0.6094	0.4707	0.5332
Ref. [66]	102	0.0781	254	0.0469	0.4219	0.6406	0.4766	0.5332
Ref. [51]	104	0.1328	253	0.0391	0.4063	0.5938	0.4668	0.5430
Ref. [12]	106	0.148	255	0.0470	0.4063	0.6250	0.4710	0.5390
Ref. [48]	106	0.148	254	0.0390	0.4220	0.5940	0.4710	0.5330

**Table 3:** Comparison of NPCR results due to the proposed algorithm and some other schemes, where the bold value shows that the corresponding image passed the test.

File Name	Proposed	Ref. [71]	Ref. [14]	Ref. [28]	Ref. [15]	Ref. [16]	Ref. [17]	Ref. [18]	Ref. [57]	Ref. [51]	Ref. [70]
5.1.10	**99.5911**	**99.6095**	**99.6353**	**99.6154**	99.5513	**99.5803**	**99.6459**	**99.6397**	**99.6094**	**99.6399**	**99.61**
5.1.11	**99.6155**	**99.6133**	**99.6277**	**99.6244**	99.53	**99.6215**	**99.5803**	**99.6018**	**99.5926**	99.5605	**99.64**
5.1.12	**99.6292**	**99.6123**	99.5351	**99.5703**	**99.5789**	**99.6231**	**99.6154**	**99.6321**	**99.6063**	**99.5972**	**99.60**
5.1.13	**99.5972**	**99.6050**	**99.617**	**99.6109**	99.2706	**99.5971**	99.44	**99.6351**	**99.6201**	**99.6201**	**99.63**
5.1.14	**99.6323**	**99.6210**	**99.6109**	**99.6364**	**99.5986**	**99.6353**	**99.5803**	**99.6063**	**99.5941**	**99.6017**	**99.62**
7.1.02	**99.6277**	**99.6117**	**99.6124**	**99.6075**	99.4747	**99.6097**	99.5544	**99.6584**	**99.6044**	**99.6021**	**99.62**
7.1.06	**99.6376**	**99.6064**	**99.6078**	**99.6272**	99.5506	**99.6086**	**99.5925**	**99.6291**	**99.6147**	**99.6346**	**99.61**
5.3.01	**99.6292**	**99.6095**	**99.6099**	99.5931	99.5977	**99.6242**	**99.6091**	**99.6128**	**99.6024**	**99.6059**	**99.60**
5.3.02	**99.6571**	**99.6095**	**99.6076**	**99.6128**	99.5534	**99.6125**	**99.6033**	**99.6159**	**99.6100**	**99.6027**	**99.62**
Pass/All	9/9	9/9	8/9	8/9	2/9	9/9	7/9	9/9	9/9	8/9	9/9

**Table 4:** Comparison of UACI results due to the proposed algorithm and some other schemes, where the bold value shows that the corresponding image passed the test.

File Name	Proposed	Ref. [71]	Ref. [14]	Ref. [28]	Ref. [15]	Ref. [16]	Ref. [17]	Ref. [18]	Ref. [57]	Ref. [51]	Ref. [70]
5.1.10	**33.3765**	**33.4663**	**33.4478**	**33.3640**	30.1968	33.6559	32.4913	**33.3592**	**33.2932**	33.2502	33.24
5.1.11	**33.4904**	**33.4554**	**33.5105**	**33.5293**	31.7477	33.2149	32.9639	**33.4603**	**33.3983**	**33.3431**	33.72
5.1.12	**33.5736**	**33.4604**	**33.4483**	**33.3835**	**33.5818**	**33.3513**	**33.4799**	**33.4650**	**33.3457**	**33.2988**	33.56
5.1.13	**33.4112**	**33.4601**	**33.5006**	**33.4355**	40.1144	**33.4222**	**33.5458**	**33.5112**	**33.2842**	**33.3081**	33.77
5.1.14	**33.5087**	**33.4606**	**33.4946**	**33.4754**	30.0463	**33.5030**	32.6501	**33.3569**	**33.3674**	33.2394	33.21
7.1.02	**33.3974**	**33.4563**	**33.5150**	**33.5432**	29.3539	**33.4345**	31.9622	**33.4765**	33.2943	33.2690	**33.53**
7.1.06	**33.4346**	**33.4515**	**33.3860**	**33.5144**	29.8338	33.4610	32.3346	**33.3988**	**33.3885**	33.3408	33.30
5.3.01	**33.4680**	**33.4511**	**33.4744**	**33.4981**	32.4783	**33.4344**	33.0525	**33.4723**	33.3002	33.3506	**33.42**
5.3.02	**33.4337**	**33.4536**	**33.4877**	**33.4800**	30.4249	**33.4542**	32.6017	**33.4906**	33.3224	33.3033	33.29
Pass/All	9/9	9/9	9/9	9/9	1/9	6/9	2/9	9/9	6/9	3/9	2/9

**Table 5:** Comparison of entropy results due to the proposed algorithm and some other schemes.

File Name	Proposed	[14]	[28]	[15]	[16]	[17]	[18]	[19]	[20]	[57]	[51]	[46]	[72]	[41]
Lena	7.9994	7.9993	7.9993	7.9634	7.9992	7.9976	7.9994	7.9993	7.9972	7.9993	7.9991	7.9894	7.9993	7.9994
Barbara	7.9993	7.9994	7.9992	7.9667	7.9993	7.9979	7.9993	7.9992	-	7.9991	7.9993	-	7.9993	7.9994

**Table 6:** Comparison of correlation results along all the three directions for the Lena and Barbara images, due to the proposed algorithm and some other schemes.

Scheme	Lena			Barbara		
	Horizontal	Vertical	Diagonal	Horizontal	Vertical	Diagonal
Proposed	−0.0006	−0.00009	−0.0005	0.0007	0.0014	−0.0005
Ref. [14]	−0.0026	−0.0054	0.0082	-	-	-
Ref. [28]	−0.0353	0.0286	−0.0249	-	-	-
Ref. [15]	−0.0011	−0.0020	0.0064	-	-	-
Ref. [16]	0.0027	0.0003	0.0012	0.0005	0.0068	0.0003
Ref. [17]	−0.0005	−0.0011	−0.0015	-	-	-
Ref. [18]	0.0039	0.0059	−0.0050	-	-	-
Ref. [19]	−0.0013	0.0080	−0.0094	−0.0047	0.0007	0.0060
Ref. [20]	−0.0005	0.0012	0.0007	-	-	-
Ref. [51]	0.0009	0.0097	−0.0013	−0.0016	0.0038	0.0014
Ref. [57]	−0.0003	−0.0005	0.0005	−0.0002	0.0003	−0.0006
Ref. [46]	0.0023	0.0029	0.0021	-	-	-
Ref. [72]	0.0019	−0.0024	0.0011	-0.0024	0.0031	−0.0013
Ref. [41]	0.0019	−0.0006	−0.0014	−0.00007	−0.0022	0.0007

**Table 7:** Analysis of the proposed encryption technique against plain-text attacks.

Plain Image	NPCR (%)	UACI (%)	Correlation of Cipher Image			Entropy
			Hori.	Ver.	Diag.	
All-black	99.62	33.42	0.0046	0.0036	−0.0040	7.9974
All-white	99.62	33.49	0.0062	0.0037	0.0012	7.9974

**Table 8:** The NPCR, UACI and entropy results of the encrypted color images.

Image	Size	NPCR (%)			UACI (%)			Entropy		
		R	G	B	R	G	B	R	G	B
Female	256×256	99.61	99.57	99.64	33.32	33.33	33.28	7.9974	7.9972	7.9971
Lena	512×512	99.61	99.66	99.63	33.41	33.45	33.44	7.9992	7.9992	7.9994
San Francisco	1024×1024	99.67	99.67	99.73	33.48	33.45	33.48	7.9998	7.9998	7.9998

**Table 9:** Comparison of NPCR and UACI results for the color image of Lena${}_{512\times 512}$.

Scheme	Image	NPCR (%)				UACI (%)			
		R	G	B	Average	R	G	B	Average
Proposed	Lena	99.61	99.66	99.63	99.64	33.41	33.45	33.44	33.44
Ref. [73]		99.63	99.62	99.65	99.63	33.60	33.50	33.55	33.55
Ref. [74]		99.62	99.64	99.60	99.62	33.43	33.45	33.43	33.44
Ref. [75]		99.56	99.63	99.63	99.61	35.46	33.22	33.02	33.90
Ref. [76]		99.63	99.63	99.57	99.61	33.49	33.38	33.47	33.45
Ref. [77]		99.61	99.61	99.61	99.61	33.43	33.50	33.38	33.43
Ref. [28]		99.62	99.63	99.59	99.61	33.44	33.53	33.48	33.48
Ref. [29]		99.60	99.60	99.60	99.60	33.55	33.50	33.46	33.50
Ref. [78]		99.69	99.70	99.50	99.63	33.36	33.58	33.38	33.44
Ref. [7]		99.61	99.62	99.61	99.61	33.51	33.45	33.39	33.45

**Table 10:** Comparison of the Entropy results for the color image of Lena${}_{512\times 512}$.

Scheme	Image	Entropy			
		R	G	B	Average
Proposed	Lena	7.9992	7.9992	7.9994	7.9993
Ref. [73]		7.9994	7.9993	7.9993	7.9993
Ref. [74]		7.9912	7.9914	7.9915	7.9914
Ref. [75]		7.9278	7.9744	7.9705	7.9576
Ref. [76]		7.9992	7.9993	7.9994	7.9993
Ref. [77]		7.9895	7.9894	7.9894	7.9894
Ref. [28]		7.9993	7.9993	7.9993	7.9993
Ref. [29]		7.9993	7.9992	7.9993	7.9993
Ref. [78]		7.9992	7.9994	7.9993	7.9993
Ref. [7]		7.9993	7.9994	7.9994	7.9994
Ref. [43]		7.9993	7.9994	7.9993	7.9993

**Table 11:** Correlation coefficients of two adjacent pixels in encrypted color images.

Image	Size	Correlation								
		Horizontal			Diagonal			Vertical		
		R	G	B	R	G	B	R	G	B
Female	256×256	−0.00177	0.00240	0.00253	0.00143	−0.00256	−0.00354	−0.00097	0.00113	−0.00027
Lena	512×512	0.00035	−0.00221	0.00084	0.00108	0.00002	0.00007	0.00200	0.00133	−0.00026
San Francisco	1024×1024	−0.00059	−0.00067	0.00125	0.00083	−0.00013	0.00002	0.00158	−0.00031	0.00120

## Abbreviations

The following abbreviations are used in this manuscript:

ECs	Elliptic curves
S-box	Substitution box
